# Integrative Approaches for microRNA Target Prediction: Combining Sequence Information and the Paired mRNA and miRNA Expression Profiles

**DOI:** 10.2174/1574893611308010008

**Published:** 2013-02

**Authors:** Su Naifang, Qian Minping, Deng Minghua

**Affiliations:** 1 LMAM, School of Mathematical Sciences, Peking University, Beijing 100871, P.R. China; 2 Beijing International Center for Mathematical Research, Peking University, Beijing 100871, P.R. China; 3 Center for Theoretical Biology, Peking University, Beijing 100871, P.R. China; 4 Center for Statistical Sciences, Peking University, Beijing, P.R. China

**Keywords:** Expression profile, integrative analysis, miRNA, target prediction.

## Abstract

Gene regulation is a key factor in gaining a full understanding of molecular biology. microRNA (miRNA), a novel class of non-coding RNA, has recently been found to be one crucial class of post-transactional regulators, and play important roles in cancer. One essential step to understand the regulatory effect of miRNAs is the reliable prediction of their target mRNAs. Typically, the predictions are solely based on the sequence information, which unavoidably have high false detection rates. Recently, some novel approaches are developed to predict miRNA targets by integrating the typical algorithm with the paired expression profiles of miRNA and mRNA. Here we review and discuss these integrative approaches and propose a new algorithm called HCTarget. Applying HCtarget to the expression data in multiple myeloma, we predict target genes for ten specific miRNAs. The experimental verification and a loss of function study validate our predictions. Therefore, the integrative approach is a reliable and effective way to predict miRNA targets, and could improve our comprehensive understanding of gene regulation.

## INTRODUCTION

Discovering gene regulation is one of the main goals in molecular biology. Specifically, uncovering the mechanisms underlying the expression of tumor related genes is a key factor in gaining a full understanding of cancer biology [1], which is also of great therapeutic significance.

While previously a great deal of study has focused on transcriptional factors (TFs), one crucial class of regulators at the transcriptional level, the post-transcriptional regulator microRNA (miRNA) has arrested much attention recently. miRNAs are a noval class of endogenous ~22nt noncoding RNAs. They down regulate gene expression through the following procedures. First, the primary miRNA are transcribed from “miRNA genes” or spliced from the intronic regions of their host genes. Then the primary miRNAs produce the miRNA precursors, and the final mature miRNAs. These miRNAs are combined with Argonaute (Ago) proteins to form RNA-induced silencing complexes (RISCs). RISCSs bind to the 3’- untranslated region of target mRNAs, which lead to their translational repression or degradation [2]. Hundreds of miRNAs have been annotated in human genome, and they are predicted to regulate up to one third of all protein-coding genes [3].

Experimental analysis has recognized that miRNAs control the key cellular processes such as growth, development and apoptosis [4]. It has been established that miRNAs make an important contribution to gene regulation in embryonic development and human disease, especially cancer [5-8]. Previous studies have verified that miRNAs can act as tumor suppressors or oncogenes and their dysregulation is widely involved in cancer initiation and progression [9], which enables their inhibition to be a novel therapeutic strategy for cancer [10].

An essential step and major challenge in understanding miRNA regulatory function is the identification of their target genes [11]. Since it is infeasible to carry out high thoughput experiments, only a small fraction of miRNA targets have experimental supports [12, 13]. Typically, the target prediction is achieved by computational approaches based on sequence analysis. A great deal of target prediction programs have been developed [14-18]. Among them, TargetScan [3, 19], PicTar [20] and miRanda [21] are the most common ones. Generally, they use the following principles to recognize miRNA targets: 1) seed match: the 6-8nt seed in miRNA 5’ part pair to the 3’ UTR region of their target mRNA; 2) thermodynamic stability: the free energy of the miRNA-mRNA hybrid is low; 3) conservation: miRNA target sites are conserved among several species. However, these sequence based approaches have high false-positive rate. It has been demonstrated that the false positive rate of TargetScan prediction is about 22-31% [22]. Since the seed match complementation could not discern the real targets effectively, great deals of fake targets are confounded.

With the development of high throughput technology, more and more miRNA and mRNA expression profiles have been achieved to investigate miRNA’s role in biological processes, especially cancer [9, 23]. Previous studies have revealed that miRNA greatly repress their target mRNAs, and mRNAs have significant expression changes after miRNA transfection or inhibition. It has also been verified that the expression of mRNAs targeted by highly expressed miRNAs are negative shifted compared with other mRNAs. Therefore, the significantly negative correlated miRNA-mRNA pairs have high potential to be the real target pairs [24].

Based on this idea, some novel strategies have been developed to predict miRNA targets by integrative analysis. They mainly use the paired miRNA and mRNA expression data, which profile miRNA and mRNA expression levels simultaneously from the same sample, to supplement the sequence prediction for the detection of actual miRNA targets.

In this article, we review and discuss the most recent integrative approaches for miRNA target predictions. We also develop a new method called HCtarget. We apply HCtarget to the expression data in multiple myeloma and evaluate the performance of our predictions.

## REVIEW OF PREVIOUS APPROACHES

In the recent developed integrative approaches, there are roughly three ways to incorporate miRNA and mRNA expression profiles to the sequence prediction (Table **1**): 1) directly consider the correlation between miRNA and mRNA expression; 2) formulate mRNA and miRNA expression with linear model with latent varialbes 3) use Bayesian network to model the miRNA-mRNA regulatory network.

## CORRELATION BASED APPROACH

Since miRNA generally repress their target mRNAs, a straightforward way to validate miRNA targeting mRNAs is detecting whether their expressions are inversely correlated. Based on this idea, some recent approaches have been developed to integrate the correlations between miRNA and mRNA pairs for the target predictions.

MMIA [25] (miRNA and mRNA Integrated Analysis) is an integrated miRNA and mRNA analyzing web server. It incorporates the common miRNA target prediction algorithms TargetScan, PITA and PicTar, and restricts the predictions on the significantly up (down) expressed miRNAs and the corresponding down (up) expressed mRNAs. MMIA is a feasible and simple tool for integrating miRNAs and mRNA expression profiles. However, it only takes into account the significantly up and down expression features, and loses the information of their whole expression patterns and their correlations.

X. Peng *et al.* [26] develop this approach by considering the inverse expression relationships between miRNAs and mRNA. They calculate the Pearson correlations between every miRNA-mRNA pair, and select the significant inverse expression pairs to construct a binary miRNA-mRNA correlation network. Meanwhile, they build a miRNA-mRNA target network based on sequence analysis. Here they relax the prediction criteria to the seed match principle, without demanding phylogenetic conservation or thermodynamic stability, to provide a larger set of candidate targets. Finally, the correlation network and the target prediction network are intersected to provide an integrative miRNA-mRNA regulatory network. This approach proposes a new point of view for miRNA target prediction, which replaces some sequence criteria by the inverse expression relationships.

G. Huang *et al.* provide mirConnX [27], a web interface for inferring and displaying mRNA and miRNA regulatory network. It combines five prediction algorithms including PITA, miRANDA, TargetScan, RNAhybrid and Pictar to achieve an integrative target prediction score between each miRNA-mRNA pair. The experimental verified miRNA targets [12] are also incorporated. Meanwhile, mirConnX integrates the miRNA-mRNA expression profiles by calculating the correlations (Pearson, Spearman or Kendall) between miRNA-mRNA pairs. These correlations are converted to the probabilities of association. The target scores and the association probabilities are weighted summed to the final prediction scores, with a user defined weight. mirConnX has two innovations. First, besides Pearson correlation, it considers the non-parametric coefficients (Spearman or Kendal) and converts them to probabilities. When the sample size is small or there are outliers in the expression data, this correlation is more reliable. Second, the correlation network and the target network are weighted integrated instead of the simple intersecting.

MAGIA [28] (miRNA and genes integrated analysis) is a similar web tool for the integrative analysis. It extracts the target predictions from miRanda, PITA and TargetScan, and provide four approaches to integrate miRNA and mRNA expression profiles. 1)Similar to mirConnX, compute the Pearson or Spearman correlation coefficients between each predicted miRNA-mRNA pairs, and convert them to a false discovery rate. 2) Calculate the mutual information between a miRNA expression and a mRNA expression based on nearest neighbor distance. It could be regarded as a generalization of the Pearson correlation. 3) GenmiR++, which would be described in the following part. 4) Meta-analysis when miRNA and mRNA profiles are not paired. Users could select one or several approaches and take the intersection or union to display the combined regulatory network.

S. Bandyopadhyay *et al.* propose a new point of view to integrate the expression data [29]. Their approach TargetMiner is a support vector machine (SVM) classifier for miRNA target prediction. It incorporates expression profiles to construct a reliable training set. Previously, the training set are putatively extracted from experimentally verified miRNA targets (from Tarbase [12] and miRecords [13]), or sequence based predictions (from miRanda, TargetScanS, PicTar and DIANA-microT). However, the number of verified targets is pretty small, and the predictions have a significant number of false positive targets. TargetMiner propose a multi-stage filtering approach to identify the non-targets in these predictions. It first identifies tissue specific miRNAs and mRNAs by analyzing miRNA and mRNA expression profiles across several tissues, and then selects mRNA as non-argets if it is over-expressed in the same tissue with its corresponding miRNA. These candidate non-targets are further filtered by removing mRNAs with feasible miRNA-mRNA duplex stability or seed-site conservation. Combining the experimentally verified miRNA targets, TargetMinner achieve an integrative training data of miRNA targets and non-targets. A SVM classification model is built on this data, with 30 features extracted and selected from sequence site context information. The learned SVM classifier could efficiently predict miRNA targets. Generally, TargetMinner provide an integrative training data for learning a classifier. However, it only considering the expression pattern in the training procedure, without taking them as the classification features in the SVM model.

E. Gammazon *et al.* develop a new approach ExprTarget [30] by combining the sequence prediction approach and the expression features in the classification. Focus on a certain miRNA, ExprTarget constructs a logistic model as:

$$\mathrm{logit}\left(p_{i}\right)=\left.\log\left(\frac{p_{i}}{1-p_{i}}\right)\right|=\beta_{0}\beta_{p}X_{i}^{p}+\beta_{t}X_{i}^{t}+\beta_{m}X_{i}^{m}+\beta_{e}X_{i}^{e}$$

Here *p_i_* is the probability that mRNA *i* is a real target. $X_{i}^{p}$, $X_{i}^{t}$ and $X_{i}^{m}$ are the target prediction scores of mRNA *i* from Pictar, TargetScan and miRanda respectively. $X_{i}^{e}$ is expression feature, defined as the p value of the general linear model between mRNA *i* and the miRNA. Note that if the estimated coefficient in the model is positive, $X_{i}^{e}$ is set to 1. The coefficients *β* describe the contribution weights of different prediction algorithms. Extracting the experimental validate miRNA targets as training data, *β* could be estimated using logistic regression. By this means, ExprTarget provide the target probabilities for each mRNA. ExprTarget extends TargetMinner by incorporate the expression features in the classifier. This feature $X_{i}^{e}$ could be regarded as a generalization of the Pearson correlation, so ExprTarget is also an extension of mirConnX, with the weights learned from experimental validate targets.

Beside the above approaches, there are some other web tools that combine miRNA-mRNA expression profiles with their target predictions. miRGator [31] integrates miRanda, PicTar and TargetScan target predictions, and displays the expression correlations between miRNA-mRNA pairs. The rank list of target mRNAs sorted by their correlations with the corresponding miRNA could also be provided. MirZ [32] incorporates smiRNAdb, a database containning miRNA sequencing profiles, and the ElMMo miRNA target prediction algorithm. It also integrates mRNA expression data and allow user to restrict the target prediction to specific mRNAs that expressed in a given cell type. mimiRNA [33] integrates expression data from human miRNAs and mRNAs across multiple tissues or cell types. It groups and separates miRNA or mRNA expression data into several tissues and cell types. The paired expression data could be visualized. mimiRNA also incorporates TargetScan, miRBase, RNA22 and PicTar. User could search the targets and the inverse expressed mRNAs for a given miRNA.

In addition, when miRNA expression data are not available, HOCTAR [34] (host gene oppositely correlated targets) could be employed. It considers that most human miRNAs are intragenic and are transcribed as part of their hosting transcription units, so the expression of miRNA host genes could be used as a proxy of the expression of the miRNA itself. Based on this idea, HOCTAR extracts a great deal of mRNA expression profiles and provides an average inverse correlated score between each mRNA and miRNA host gene pair. These scores are then integrated with the miRanda, TargetScan, and PicTar predictions.

## LINEAR MODEL APPROACH

The previous approaches only consider the pairwise expression correlation between miRNA and mRNA. However, mRNA may be regulated by multiple miRNAs and its expression is affected synthetically by all the targeting miRNAs. Based on this idea, some novel methods have been developed to model miRNA’s combinatorial effect on their target mRNAs.

Among them, GenmiR++ (Generative model for miRNA regulation) is the most widely used approach [24, 35]. It characterizes mRNA expressions as a linear combination of the regulatory effects of their targeting miRNAs, and a variational Bayesian algorithm is used to learn the latent miRNA target indicators. It has been successfully applied on the paired miRNA and mRNA expression data among multiple tissues.

Let *y_it_* denote the expression level of mRNA *i* in tissue *t* and *z_jt_* denote the expression level of miRNA *j* in the same tissue, where *i* = 1, …, N, *j* = 1, …, *M* and *t* = 1, …, *T*. GenMiR++ take a linear model to formulate the mRNA expressions and the regulatory effects of their targeting miRNAs. A latent binary variable *R* is used to indicate the target relations, where *r_ij_* = 1 if mRNA *i* is targeted by miRNA *j*, and 0 otherwise. The relationship between mRNA and miRNA expressions is formulated as:

$$y_{\mathrm{it}}=\mu_{t}-\gamma_{t}\sum_{j=1}^{M}\lambda_{j}r_{\mathrm{ij}}z_{\mathrm{jt}}+\varepsilon_{\mathrm{it}}$$

or

$${\vec{y}}_{i}=\vec{\mu }-\sum_{j=i}^{M}\lambda_{j}r_{\mathrm{ij}}\Gamma {\vec{z}}_{j}+{\vec{\varepsilon }}_{i},{\vec{\varepsilon }}_{i}∼N\left(0,\Sigma \right)$$

here *λ_j_* represents the regulatory effects of miRNA *j*, *γ_t_* accounts for the expression scaling in tissue *t*, and µ_t_ is the background effect of tissue *t*.

The latent variable *R* indicates the target relations between miRNA and mRNA. Integrating the target predictions *C* from TargetScan, as *c_ij_* = 1 if mRNA *i* is predicted to be targeted by miRNA *j*, and 0 otherwise, GenmiR++ assign *R* a Bernoulli distribution depend on *C*. That is *r_ij_* ~ *bernoulli* (π) in the condition of *r_ij_* = 1, and *r_ij_* = 0 when *c_ij_* = 0.

Assigning the prior as *γ_t_* ~ *N* (1, *s*^2^) and λ_j_ ~ exp(α), GenmiR++ use a variational Bayesian algorithm to estimate the posterior distribution of *r_ij_*. Since its form is complicated, instead of learning the real posterior, the variational Bayesian algorithm provide a factorized variational posterior for approximation [40]. By this means, the computation is simplified and the target probability could be achieved.

GenMiR++ has also been developed to GenmiR3 [41], with an alternative prior distribution and the parameter π is modified by integrating the sequence information such as the hybridization energy and context score.

GenMiR++ has been widely used to integrate the miRNA-mRNA expression data with the target predictions. However, it has several restrictions. First, originating from the experiments of different tissues, GenmiR++ characterizes miRNA’s relative effects among all tissues as a constant. This assumption may not hold when considering the experiments of different cancer patients. Since patients have much more varieties, their miRNA’s relative effects could not be regarded as a constant anymore. Second, GenMiR++ uses variational Bayesian algorithm to learn the parameters. The variational posterior may deviated from the real posterior. Its convergence rate is highly depends on the form of the likelihood and priors and may be extremely slow.

F. Stingo *et al.* [36] propose a similar linear approach. Different from GenmiR++, they don’t take into account the tissue effect, and consider that miRNA has distinct regulatory effect on differet mRNAs. Based on this idea, they propose a linear model to fomulate miRNA and mRNA expressions:

$$y_{i}=\sum_{j=1}^{M}\beta_{\mathrm{ij}}r_{\mathrm{ij}}z_{j}+\varepsilon_{i},\varepsilon_{i}∼N\left(0,\sigma_{i}^{2}\right),i=1,....,N$$

here *y_i_* is the expression of mRNA *i* and *z_j_* is the expression of miRNA *j*. *β_ij_* represents the effect of miRNA *j* on mRNA *i*, in GenmiR++ this term is uniformed to *λ_j_*. Meanwhile, the target indicator *r_ij_* is assigned with Bernoulli distribution, with a modified parameter:

$$\pi_{\mathrm{ij}}=\frac{\exp\;\left[\eta +\tau_{1}c_{\mathrm{ij}}^{1}+\tau_{2}c_{\mathrm{ij}}^{2}+\tau_{3}c_{\mathrm{ij}}^{3}+\tau_{4}c_{\mathrm{ij}}^{4}+\tau_{5}c_{\mathrm{ij}}^{5}\right]}{1+\exp\;\left[\eta +\tau_{1}c_{\mathrm{ij}}^{1}+\tau_{2}c_{\mathrm{ij}}^{2}+\tau_{3}c_{\mathrm{ij}}^{3}+\tau_{4}c_{\mathrm{ij}}^{4}+\tau_{5}c_{\mathrm{ij}}^{5}\right]}$$

where $C_{\mathrm{ij}}^{1}$ , $C_{\mathrm{ij}}^{2}$ , $C_{\mathrm{ij}}^{3}$ , $C_{\mathrm{ij}}^{4}$ , and $C_{\mathrm{ij}}^{5}$ , are the prediction scores of PicTar, miRanda, aggregate TargetScan, total TargetScan and PITA respectively.

With the prior *β_ij_* ~ *Gamma* (1, *cσ*_i_) and σi−1∼Gamma δ+M2,d2 , the posterior distribution could be estimated using Metropolis-Hasting algorithm. Thus the posterior target scores $p\left(r_{\mathrm{ii}}=1|\mathrm{data}\right)$ are achieved to construct miRNA regulatory network.

However, since *α* are distinct for different miRNAs and mRNAs, the model has a great deal of parameters. Therefore, this approach is limited in high computational complexity.

J. Li *et al.* [37] also modify the model. They discretize mRNA expression to binary value *y_it_* = 1 or 0, which represent high or low expressions, then assume *y_it_* follow a logistic model: let *q_it_* = P(*y_it_* = 1),

$$\log\left(\frac{q_{\mathrm{it}}}{1-q_{\mathrm{it}}}\right)=\sum_{j=1}^{M}\phi_{j}r_{\mathrm{ij}}z_{\mathrm{jt}}+\varepsilon_{t},i=1,....,N$$

Similar to GenmiR++, *r_ij_* follow a bernoulli distribution depend on the TargetScan prediction *c_ij_* with parameter π.

With the prior *ф_j_* ~ exp(*ф*),*ф* ~ *U*(0,∞), *ε_t_* ~ *U*(-50, 50) and *ф* ~ *beta*(1, 1), the posterior could be estimated using Gibbs sampling. They also apply the similar approach to study the relation between miRNA expression and protein abundance.

In this approach, the binary mRNA values lose the information of the whole expression profile.

The above approaches use Bayesian methodology for parameter estimation. On the other hand, Y. Lu *et al.* [38] incorporate a lasso regression model to predict miRNA targets. Moreover, they pay attention to the role of RISCs and assume that mRNA expression follow a linear model with its targeting RISCs. The RISC level could be obtained through the expression of its comprising miRNA and Ago proteins. There are four Ago proteins in human, and Ago2 is the essential one. Therefore, the model is:

$$y_{i}=\beta_{\mathrm{i0}}+\sum_{j=1}^{M}c_{\mathrm{ij}}\beta_{\mathrm{ij}}z_{j}{\mathrm{Ago}}_{2}+\sum_{j=1}^{M}c_{\mathrm{ij}}\phi_{\mathrm{ij}}z_{j}{\mathrm{Ago}}_{134}+\varepsilon_{i}$$

Here *y_i_* is the expression of mRNA *i*, *z_j_* is the expression of miRNA *j*, *Ago*_2_ is the expression of Ago2 mRNA and *Ago*_134_ is the combined expression of Ago1, Ago3 and Ago4 mRNA. *c_ij_* indicates the target prediction relation from TargetScan and PicTar.

Then a multi-run lasso regression procedure is produced, and miRNAs are ranked by their estimated coefficients. With these ranked scores, the targeting miRNAs could be achieved.

However, this approach produces lasso regression for each mRNA separately. It will be time consuming when applying to a great deal of mRNAs.

## BAYESIAN NETWORK APPROACH

Beside the linear model approach, some novel studies are developed to model the whole miRNA-mRNA regulatory network. Bayesian network, a probabilistic graphical model, has been widely used to discover the structure of gene networks [42]. It could also be applied to study the regulation between miRNA and mRNA [43]. Liu *et al.* [39] develop a new approach which use Bayesian network to learn the miRNA-mRNA regulatory network by integrating miRNA target prediction and expression profiles.

Denote miRNA and mRNA as nodes and their target relations as directed edges, the regulatory network could be modeled as a discrete Bayesian network *G*. The miRNA and mRNA expressions *X* are discretized to binary values 1 and 0, indicating high and low expressions. Let *N_ijk_* be the observed times that mRNA *X_i_* is in state *k* (*k* = 1, …, *r_i_*, here *r_i_* = 2) with its parent miRNAs in state *j* (*j* = 1, …, *q_i_*), then *X* follow a multinomial distribution with parameter *θijk* = *P* (*X_i_* = *k*|*parent*(*X_i_* ) = *j*):

$$p\left(X|\theta G\right)\propto \prod_{i=1}^{n}\prod_{j=1}^{\mathrm{qi}}\prod_{k=1}^{\mathrm{ri}}\theta_{\mathrm{ijk}}^{N_{\mathrm{ijk}}}$$

Assigning the Dirichlet prior to *θ* as

$$p\left(\theta |G\right)\propto \prod_{i=1}^{n}\prod_{j=1}^{\mathrm{qi}}\prod_{k=1}^{\mathrm{ri}}\theta_{\mathrm{ijk}}^{N_{\mathrm{ijk}}-1}$$

the Bayesian score of the network *p*(*X*|*G*) is given by [44]:

$$p\left(X|G\right)=\prod_{i=1}^{n}\prod_{j=1}^{q_{i}}\frac{\Gamma \left(\alpha_{\mathrm{ij}}\right)}{\Gamma \left(N_{\mathrm{ij}}+\alpha_{\mathrm{ij}}\right)\Gamma }\prod_{k=1}^{r_{i}}\frac{\Gamma \left(N_{\mathrm{ijk}}+\alpha_{\mathrm{ijk}}\right)}{\Gamma \left(\alpha_{\mathrm{ijk}}\right)}$$

Here $N_{\mathrm{ij}}=\sum_{k=1}^{r_{i}}N_{\mathrm{ijk}},\alpha_{\mathrm{ij}}=\sum_{k=1}^{r_{i}}\alpha_{\mathrm{ijk}}$ and *α_ijk_* = *N* / *r_i_q_i_*, *N* is the sample size.

Network with the maximum score is selected as the learned Bayesian network, which is putatively achieved by exhaustive searching algorithm such as hill climbing. The searching space could be reduced by constraining the target relations within miRBase, PicTar and TargetScan predictions. By this means, Liu *et al.* analyze miRNA-mRNA expression profiles from multiple cell types and build Bayesian network for each cell type. These networks are then integrated to provide the significant miRNA-mRNA target relations.

Bayesian network is a reliable and accurate model for the regulatory network [42]. However, its learning algorithm has high computational complexity and is time consuming. Therefore, Bayesian network could not be applied to learn large-scale networks.

## HCTARGET METHOD

Based on the above discussion, we propose a new algorithm called HCtarget (High Confident targets) to integrate expression and sequence information to detect miRNA targets. Our approach extends GenMiR++ and overcomes its restrictions in the following two ways. First, GenmiR++ characterizes miRNA’s relative effects among all tissues as a constant. We improved this constrain by re-defining the parameters of miRNA effects. Second, GenMiR++ uses variational Bayesian algorithm to approximate of the real posterior. Its convergence rate may be slow and the estimation is not stable. We use a classical Markov chain Monte Carlo (MCMC) algorithm to learn the posterior directly.

## MODEL

Incorporating the notations in GenmiR++, we propose a linear model to formulate the relations between mRNA expressions and the regulatory effects of their targeting miRNAs as:

$$y_{\mathrm{it}}=\beta_{0t}+\sum_{j=1}^{M}r_{\mathrm{ij}}z_{\mathrm{jt}}\beta_{\mathrm{jt}}+\varepsilon_{\mathrm{it}},\varepsilon_{\mathrm{it}}∼N\left(0,\sigma_{t}^{2}\right)$$

here *β_jt_* represents the regulatory effects of miRNA *j* at sample *t* (in GenMiR++, this term is factored into the product of the tissue effect and the miRNA effect *γ_t_ λ_j_*), and β_0*t*_ is the background effect of sample *t*.

The goal of our model is to estimate the latent indicators *R*. Similarly, it follow a Bernouli distribution depend on the sequence prediction *C*. In the following discussion, we focus on the pair with *c_ij_* = 1. The likelihood of *R* is:

$$p\left(R|\pi \right)\propto \prod_{\mathrm{ij}}\pi^{c_{\mathrm{ij}}r_{j}}{\left(1-\pi \right)}^{c_{\mathrm{ij}}\left(1-r_{\mathrm{ij}}\right)}$$

here π can be regarded as the accuracy of the sequence based predictions. This assumption enables our model to cut down the false positive rate of the previous prediction.

Let *B_t_* = (*r_ij_z_jt_*), *A_t_* = [ *B_t_*], *y_t_* = (*y*_1*t*_, …., *y_Nt_*)^*T*^, *Z* = (*z_jt_*), *β*_t_ = (*β*_0*t*_, …, *β_Mt_*)^*T*^ and ∈ *_t_* = (∈_1*t*_, ...., ∈_*Nt*_)^*T*^, we have the vector representation of our model: $Y_{t}=A_{t}\beta_{t}+\in_{t}$

## MCMC ALGORITHM FOR STATISTICAL INFERENCE

Based on the above model, the likelihood of the observed data *p*(*Y*, *Z*, *C*, *R*|β, σ^2^, *ф*) is:

$$\prod_{i,t}e^{\left[{-}_{{\frac{1}{\sigma_{t}^{2}}\left(y_{\mathrm{it}}-\sum_{j=1}^{M}z_{\mathrm{jt}}r_{\mathrm{ij}}\beta_{\mathrm{jt}}-\beta_{0t}\right)}^{2}}\right]}\prod_{i,j}\pi^{c_{\mathrm{ij}}r_{\mathrm{ij}}}{\left(1-\pi \right)}^{{c_{\mathrm{ij}}}^{\left(1-r_{\mathrm{ij}}\right)}}$$

To estimate the parameters *θ* = (*β* *σ^2^*, *π*) and latent variables *R*, we apply the Bayesian methodology and a MCMC algorithm [45]. With proper prior assumptions, the posterior of *R* and *θ* have simple forms and could be directly computed using a MCMC algorithm as the following iterations [46,47]: (i) sample the parameters *θ* conditional on the updated latent variable; (ii) sample the latent variable *R* conditional on the updated parameters.

## UPDATE THE PARAMETERS

Given the non-informative prior $p\left(\beta_{t},\sigma_{t}^{2}\right)\propto \sigma_{t}^{-2}$ , the posterior distributions of *β_t_* and *σ_t_* are

$$\beta_{t}|\sigma_{t}^{2},Y∼|N\left({\hat{\beta }}_{t},{\left(A_{t}^{T}A_{t}\right)}^{-1}\sigma_{t}^{2}\right),\sigma_{t}^{2}|Y∼\upsilon s_{t}^{2}\chi_{\upsilon }^{-2}$$

where *ν* = *N* - *M* - 1 and

$${\hat{\beta }}_{t}={\left(A_{t}^{T}A_{t}\right)}^{-1}A_{t}^{T}Y_{t},{\hat{Y}}_{t}^{T}=A_{t}^{T}{\hat{\beta }}_{t},s_{1}^{2}={\frac{1}{\upsilon }\left(Y_{t}-{\hat{Y}}_{t}\right)}^{T}\left(Y_{t}-{\hat{Y}}_{t}\right)$$

While for *π*, with the conjugate prior *π ~ Beta* (*a*_0_, *b*_0_), the posterior distribution is, ~ *Beta*(*n*_1 _+ *a*_0_, *n*_0_ + *b*_0_), where $n_{1}=\sum_{\mathrm{ij}}c_{\mathrm{ij}}r_{\mathrm{ij}}$ and $n_{0}=\sum_{\mathrm{ij}}c_{\mathrm{ij}}\left(1-r_{\mathrm{ij}}\right)$

## UPDATE THE LATENT VARIABLE

The marginal distribution of the latent variable

$$p\left(r_{\mathrm{ij}}\left|c_{\mathrm{ij}}=1,Y,Z,\theta \right.\right)\propto$$

$$\exp\left[-\sum_{t-1}^{T}\frac{1}{\sigma_{t}^{2}}{\left(y_{\mathrm{it}}-\sum_{k}z_{\mathrm{kt}}r_{\mathrm{ik}}\beta_{\mathrm{kt}}-\beta_{0t}\right)}^{2}\right]\pi^{c_{\mathrm{ij}}r_{\mathrm{ij}}}{\left(1-\pi \right)}^{c_{\mathrm{ij}}\left(1-r_{\mathrm{ij}}\right)}$$

Since

$${\left[y_{\mathrm{it}}-\sum_{k}z_{\mathrm{kt}}r_{\mathrm{ik}}\beta_{\mathrm{kt}}-\beta_{0t}\right]}^{2}={\left[y_{\mathrm{it}}-\sum_{k\neq j}z_{\mathrm{kt}}r_{\mathrm{ik}}\beta_{\mathrm{kt}}-\beta_{0t}\right]}^{2}+q_{\mathrm{ijt}}r_{\mathrm{ij}}$$

here *q_ijt_* denotes

$$z_{\mathrm{jt}}^{2}\beta_{\mathrm{jt}}^{2}-{2y}_{\mathrm{it}}z_{\mathrm{jt}}\beta_{\mathrm{jt}}+2\sum_{k}z_{\mathrm{kt}}\beta_{\mathrm{kt}}z_{\mathrm{jt}}\beta_{\mathrm{jt}}r_{\mathrm{ik}}+2z_{\mathrm{jt}}\beta_{\mathrm{jt}}\beta_{0t}$$

The first term doesn’t contain *r_ij_*, so

$$p\left(r_{\mathrm{ij}}\cdot \right){\propto \exp\left(\sum_{t-1}^{T}\frac{q_{\mathrm{ijt}}}{\sigma_{t}^{2}}r_{\mathrm{ij}}\right)\pi^{c_{\mathrm{ij}}r_{\mathrm{ij}}}\left(1-\pi \right)}^{{c_{\mathrm{ij}}}^{\left(1-r_{\mathrm{ij}}\right)}}$$

that is, *r_ij_* has Bernoulli marginal distribution *p*(*r_ij_*|.) ~ *bernoulli*(*p_ij_*) with updated probability

$$p_{\mathrm{ij}}=\frac{{\left(\frac{\pi }{1-\pi }\right)}^{c_{\mathrm{ij}}}}{{\left(\frac{\pi }{1-\pi }\right)}^{c_{\mathrm{ij}}}+\exp\left(\sum_{t=1}^{T}\frac{q_{\mathrm{ijt}}}{\sigma_{t}^{2}}\right)}$$

## THE ALGORITHM OF HCTARGET

Based on the above discussion, we use a traditional MCMC approach to estimate the parameters and the latent variable iteratively:

1. Initial *β_t_*, *σ_t_*, *R* as *β_t_* = 1, *σ_t_* = 1 and *r_ij_*|*c_ij_* =1 ~ *bernoulli*(0.5) .
2. Update $\sigma_{t}^{2}$ by sampling from ${\upsilon s}_{t}^{2}\chi_{\upsilon }^{-2}$ , update *β_t_* by sampling from $N\left({\hat{\beta }}_{t},{\left(A_{t}^{T}A_{t}\right)}^{-1}\sigma_{t}^{2}\right)$ and update *π* by sampling from beta(*n*_1_ + *a*_0_, *n*_0_ + *b*_0_).
3. Given the updated parameters, sample the latent variable *r_ij_* from Bernoulli (*p_ij_*).
4. Repeat the above two steps until convergence. Here the convergence is evaluated by Gelman and Rubin criteria [47]

We output *p_ij_*, which represents the probability that miRNA *j* targets mRNA *i* given the data, for our final prediction. miRNA-mRNA pairs with *p_ij_* larger than a certain threshold are the putative target pairs of our model. In the analysis of cancer expression data, we specify the threshold as 0.8, so that our selected miRNA targets covered nearly 50% of the sequence-based predictions, and they are comparable with GenMiR++ targets.

## RESULTS

We applied HCtarget to study miRNA’s role in cancer. The computational predictions were extracted from TargetScanHuman (release 5.1). Several paired miRNA-mRNA expression datasets, such as breast cancer data (GSE19783), prostate cancer data(GSE7055) and multiple myeloma data(GSE17306) were downloaded from GEO database [48]. Since their results are similar, we took the multiple myeloma data as an example in our analysis. It profiled miRNA and mRNA expressions from 52 patients with multiple myeloma [9].

We selected multiple myeloma related miRNAs and mRNAs for our predictions. Ten miRNAs with the highest expression level were picked up, they are: hsa-let-7g, hsa-miR-142-3p, hsa-miR-148a, hsa-miR-16, hsa-miR-19b, hsa-miR-21, hsa-miR-26a, hsa-miR-29c, hsa-miR-370 and hsa-miR-494. Meanwhile 1000 mRNAs were selected, half with the highest expressions and half with the lowest expressions, since miRNA putatively repress gene expressions and may have secondary up-regulatory effects [49].

## PERFORMANCE OF HCTARGET ON THE SIMULATION DATA

First, we generated a simulation data to compare the performance of GenMiR++ and HCTarget. The ten miRNA expression data *Z* were extracted from the real data from patients with multiple myeloma, where 1000 mRNA expressions *Y* were simulated from

$$y_{\mathrm{it}}=\beta_{0t}+\sum_{j=1}^{10}r_{\mathrm{ij}}z_{\mathrm{jt}}\beta_{\mathrm{jt}}+\varepsilon_{\mathrm{it}},i=1,...1000,t=1,....,52$$

here *β_0t_*, *β_0t_* and *ε* were generated from *N*(-0.3, 0.1), *N*(1,1) and *N*(0,1) respectively. The real target relations *r_ij_* was obtained from *Bernoulli*(0.5) conditions on *c_ij_* = 1, where *c_ij_* represents the predictions in TargetScan.

Applying GenmiR++ and HCtarget on the simulation data, we computed their true positive rate and false positive rate with different cutoffs. Their ROC (Receiver operating characteristic) curves and AUC (the area under the ROC curve) values are shown in Fig. (**1**), which indicate that HCTarget has higher accuracy than GenMiR++.

## PREDICT miRNA TARGETS BASED ON CANCER EXPRESSION DATA

We then applied our HCtarget approach to the real miRNA-mRNA expression data to detect miRNA targets in cancer. TargetScan provides 1401 target pairs for our selected miRNAs and genes. HCtarget cuts down these predictions to 647, while 699 target pairs are obtained by GenMiR++.

To assess the robustness of HCtarget, we performed a series of permutation tests [24]. We permuted the gene labels 1000 times and generated 1000 random data sets. In these sets, the relationship between miRNAs and mRNAs are destroyed and their predicted target probabilities could be regarded as background. These permutations allow us to evaluate whether our model would be affected by introducing a great deal of fake targets into the candidates. Comparing the predictions of HCtarget for both permuted and original data, we found the probabilities leaned from the real data are significantly higher than the background. The p value of one side wilcoxon test is 0.1. In addition, the proportion of the probabilities bigger than 0.8 for the real data (46.2%) is higher than permuted data (44.1%). It illustrates that HCtarget could successfully discriminate the real target from the fake ones, which ensures its robustness in target prediction.

Furthermore, we extracted experimentally supported miRNA targets from Tarbase (v.5c) [12] to evaluate the accuracy of our approach. To compare Tarbase with our predictions, miRNAs were all mapped to miRNA families using the annotations in miRBase [50] For the multiple myeloma related miRNAs and mRNAs, three miRNAs and their 17 target genes have biological verifications. Nine of them are detected by HCtarget, while GenMiR++ only identifies two. The numbers of verified targets predicted by TargetScan, GenMiR++ and HCTarget as well as their precisions are listed in Table **2**, which show that HCtarget could identify more accurate targets than GenMiR++. For example, mir-15 has nine supported targets, seven of them are detected by HCtarget, while GenMiR++ failed to identify any of them. It also indicates that HCtarget has higher precision (2.78%, 18 out of 647) than the original TargetScan (2.43%, 34 out of 1401).

## VALIDATE HSA-MIR-16 TARGETS

Previous analysis suggests that hsa-miR-16 can act as a tumor suppressor in multiple myeloma [51]. We extracted a loss of function study profile of hsa-miR-16 from GEO database (GSE24522). It provided gene expression levels before and after hsa-miR-16 deletion [51]. We focused on genes with fold change larger than 1.5 as different expressed genes. For our 1000 genes, 132 genes were selected.

**Fig. (2):**
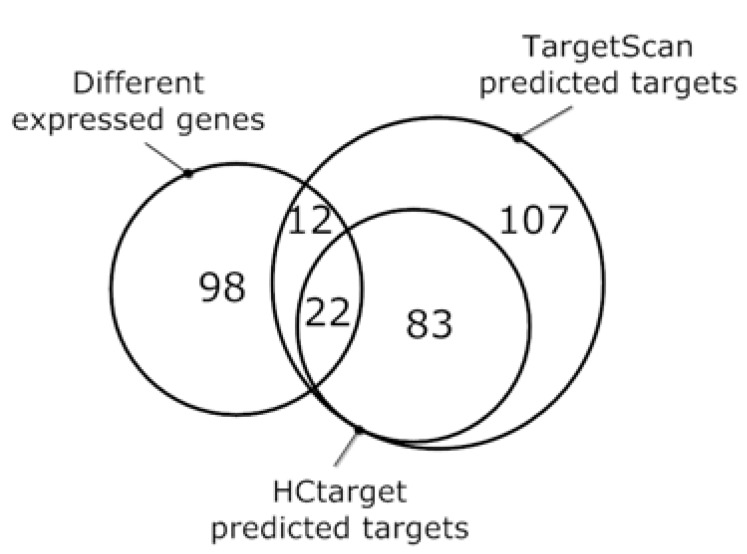
**Venn diagram**. It shows the overlap of different expressed genes with the predicted targets of targetScan and HCtarget.

To validate our prediction, we compared our detected targets with these different expressed genes. TargetScan identifies 224 targets for hsa-miR-16, 34 of them have different expression levels when hsa-miR-16 is deleted (the p value of hyper-geometric test is 0.14). HCtarget, which cuts down the target genes to 105, provides 22 validated targets (p=0.006) (Fig. **2**). This represents that HCtarget has more confirmed targets than TargetScan. In addition, GenmiR++ only detects 11 different expressed genes (p=0.72), which also validates the accuracy of HCtarget.

## GENE ONTOLOGY ENRICHMENT ANALYSIS

To have further investigation of our predicted targets, we analyzed their function annotations in Gene Ontology (GO) [52, 53]. For each miRNA target set detected by TargetScan and HCtarget respectively, we computed its GO enrichment p value using hyper geometric test. Considering multiple testing problems, these p values were corrected using FDR modification. For TargetScan, we found 107 (2.5%) functional target sets (with FDR<0.1). While there are 135 (3.1%) functional sets of GenmiR++ and HCtarget increases the number to 158 (3.7%). The comparison exhibits that the targets of HCtarget have significantly more consistent functional annotations.

Meanwhile, we selected the GO functions that significantly enriched (FDR<0.01) in hsa-miR-19b, which has been experimentally verified to be a key regulator in multiple myeloma [54]. They are: GO0034612 (response to tumor necrosis factor), GO0000723 (telomere maintenance), GO0006289 (nucleotide-excision repair), GO0006302 (double-strand break repair) and GO0045732 (positive regulation of protein catabolic process). The first annotation is significantly associated with multiple myeloma, the latter three ones are crucial functions in cell division, a key cellular process in cancer, while the last one is putative important in metabolism. These findings demonstrate that HCtarget could successfully identify the functional miRNA targets.

## EXAMPLE

**Fig. (3):**
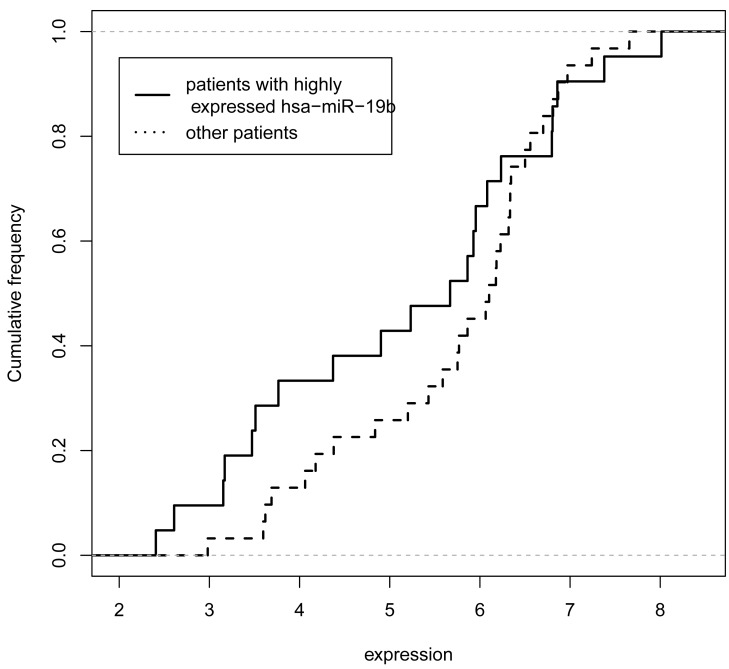
**The down regulatory effect of hsa-miR-19b on SULF1**. The cumulative distributions of the expression level of SULF1 in the sample with or without highly expressed hsa-miR-19b (red solid line and blue dashed line respectively).

Based on the above findings, we further focused on a specific target pair to discover miRNA’s role in multiple myeloma. hsa-miR-19b was selected, and one of its targets detected by HCtarget is SULF1, which has been found to be a potent inhibitor of myeloma tumor growth [55]. We focused the patients with higher hsa-miR-19b expressions (with expression level larger than average), and discovered that the expression levels of SULF1 are significantly lower in these patients than in the other ones (the p values of the one side wilcoxon test is 0.1). Their cumulative distributions (Fig. **3**) displays that the expression of SULF1 is negatively shifted when hsa-miR-19b is highly expressed. This example further confirms the significant down regulatory effects of hsa-miR-19b, and provides us a reliable target gene SULF1. We believe that this target pair plays a crucial role in multiple myeloma and could be served as effective candidates for the therapeutic treatment.

## CONCLUSION

In this paper, we review and discuss the integrative approaches that predict miRNA target genes by combining the sequence information and expression profiles.

We also propose a new algorithm, HCtarget. The simulation study and the robustness assessment confirm the accuracy of our approach. The investigations of the expression profiles in multiple myeloma also exhibit the well performance of HCtarget. Our model affords reliable targets of miRNA, which improve our understanding of miRNA’s roles in cancer. Such as the disease related target pair, hsa-miR-19b and SULF1, is beneficial for the further discovery and clinical treatment of multiple myeloma. Furthermore, selecting some other proper miRNA and mRNA expression profiles, HCtarget could be generalized to provide miRNA’s whole genome target predictions, which is helpful for the comprehensive discovering of miRNA’s regulatory effects.

Generally, the integrative approaches improve miRNA target predictions. They could be directly generalized to detect the target genes of TFs. In addition, previous studies demonstrated that TFs, or their cis-regulatory modules, have widely cooperation with miRNAs. Their combinatorial regulatory modules play important parts in gene regulation [56]. With accurate target predictions of miRNAs and TF, the integrative approaches could effectively construct gene regulatory network, which helps us to uncover the mechanisms underlying gene expression.

## Figures and Tables

**Fig. (1):**
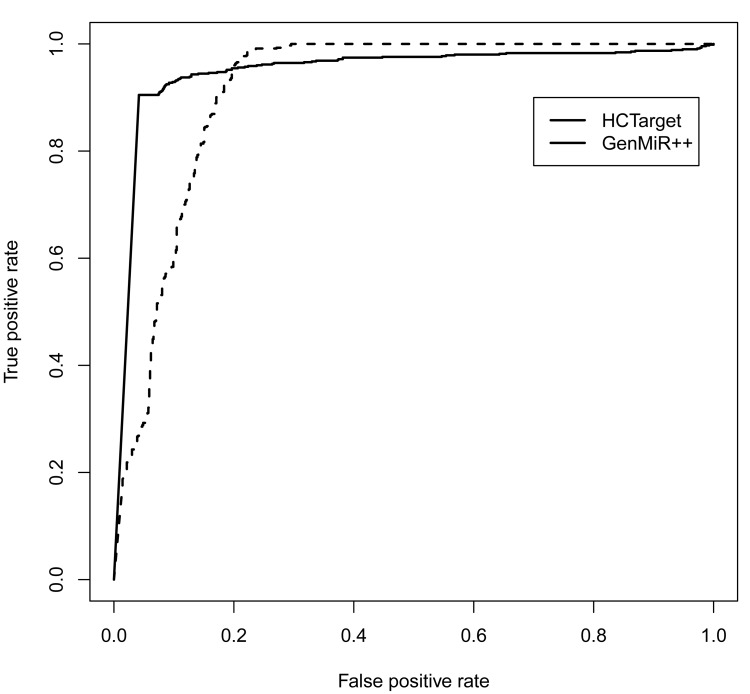
**The ROC curves of HCTarget and GenMiR++ for simulation data**. Their AUC values are 0.95 and 0.91 respectively.

**Table 1.** Integrative Approach for miRNA Target Prediction

Name	URL	Reference
**Correlation Based Approach**		
MMIA	http://cancer.informatics.indiana.edu/mmia	[25]
Peng *et al.*	-	[26]
mirConnX	http://www.benoslab.pitt.edu/mirconnx	[27]
MAGIA	http://gencomp.bio.unipd.it/magia	[28]
TargetMinner	http://www.isical.ac.in/ bioinfo_miu/	[29]
ExprTarget	http://www.scandb.org/apps/microrna/	[30]
miRGator	http://genome.ewha.ac.kr/miRGator/	[31]
MirZ	http://www.mirz.unibas.ch	[32]
mimiRNA	http://mimirna.centenary.org.au	[33]
HOCTAR	-	[34]
**Linear Mode Approach**		
GenmiR++	http://www.psi.toronto.edu/genmir/	[24, 35]
F. Stingo *et al.*	-	[36^4^]
J. Li *et al.*	-	[37]
L. Lu *et al.*	-	[38]
**Bayesian Network Approach**		
B. Liu *et al.*	-	[39]

**Table 2.** Comparison with Tarbase

miRNA Family	TargetScan	GenMiR++	HCTarget
let-7	7 (3.57%)	2 (2.02%)	2 (2.15%)
mir-15	9 (4.02%)	0 (0)	7 (6.67%)
mir-29	1 (0.51%)	0 (0)	0 (0)
total	17 (2.76%)	3 (0.95%)	9 (3.01%)
